# The students' intentions and satisfaction with the field of study and university

**Published:** 2014-10

**Authors:** ALI NOORAFSHAN, SAEEDEH POURAHMAD, MOHAMMAD MAHDI SAGHEB, ALI DEHGHANI NAZHVANI, ALI DEHSHAHRI, MANIJEH ABDOLLAHI, ZEYNAB MOHEBBI, ZAHRA KESHTKARAN, AFSANEH AHMADI, SOMAYEH KAVOUSIPOUR, FARIBA FARAHMAND, HAMID REZA KHORRAMI, ROBABEH SOLTANI, SAIED KARBALAY DOUST

**Affiliations:** 1 Histomorphometry and Stereology Research Center, Shiraz University of Medical Sciences, Shiraz, Iran;; 2 Colorectal Research Center and Biostatistics Department, Shiraz University of Medical Sciences, Shiraz, Iran;; 3 Department of Internal Medicine, Shiraz University of Medical Sciences, Shiraz, Iran;; 4 School of Dentistry, Shiraz University of Medical Sciences, Shiraz, Iran;; 5 School of Pharmacy, Shiraz University of Medical Sciences, Shiraz, Iran;; 6 School of Paramedical Sciences, Shiraz University of Medical Sciences, Shiraz, Iran;; 7 School of Nursery, Shiraz University of Medical Sciences, Shiraz, Iran;; 8 Lar Nursery School, Shiraz University of Medical Sciences, Shiraz, Iran;; 9 School of Health Sciences, Shiraz University of Medical Sciences, Shiraz, Iran;; 10 School of Rehabilitation, Shiraz University of Medical Sciences, Shiraz, Iran;; 11 School of Management, Shiraz University of Medical Sciences, Shiraz, Iran;; 12 Gerash Paramedical Sciences School, Shiraz University of Medical Sciences, Shiraz, Iran;; 13 Gifted and Talented Students Office, Shiraz University of Medical Sciences, Shiraz, Iran

**Keywords:** Medical student, Intention, Satisfaction

## Abstract

**Introduction:** The present study aimed to find an appropriate method to inform senior high school students to correctly select their academic field of study and their intentions.

**Methods:** This is a descriptive-analytic and cross-sectional study. A verified questionnaire was given to a total of 2600 students selected by stratified random sampling method (ten different colleges and entrance year from the 1^st^ to 4^th^ are considered as the strata). The position of the present field of study (major) among the list of the fields in the entrance exam was asked. The students’ methods of familiarity with different fields of study in Shiraz University of Medical Sciences (SUMS), the reasons for their selection, the students’ motivation and insistence on studying in the same field and university were asked in the questionnaire. Data were analyzed using independent two samples t-test, Analysis of Variance (ANOVA) and Chi-Square test.

**Results:** The most significant references for university field selection were high school teachers, the students' parents and the adjacency of university to one's living place. Also, the results revealed the good reputation of SUMS in the first year and its downward trend during the following years. 59.4% of the 1^st^ year students were satisfied with their field of study and SUMS. 31.8% were satisfied with the university but not with their fields of study. 6.4% were dissatisfied with the university but not with their fields of study. 2% of the students were dissatisfied with both their fields of study and university. Dissatisfaction with SUMS and field of study increased little by little so that the results obtained among the students who had entered the university earlier (in the 4^th^ year of their study) showed nearly 16.3% dissatisfaction with both the university and the study fields.

**Conclusion:** The methods for introducing the university are recommended to be revised.

## Introduction

Informative selection in each affair can have more suitable results. If the people are timely provided with correct and up to date information, they can make up their minds easily and confidently. Moreover, they will accept the results of their choice more willingly. Therefore, they will accept the future path because of their correct choice. For instance, high school students selecting their field of study in the university hope to insure their future success. Evidence has shown that a major reason for the students to go to college is “to get training for a specific career”. Nowadays, the adults who are not satisfied with their lives and careers believe one reason for their dissatisfaction to be the careless selection of and lack of enough information about their field of study in the university. A proper selection can be defined as the one which is done based on an individual's physical as well as spiritual capabilities and potential talents and this can be done just on the basis of perfect and correct information (1, 2).

In our country, Iran, university entrance exam is simultaneously held all over the country each year. In this large scale exam, there is a competition among the volunteers and they are compared in terms of their previous scientific information. Then, based on the result of the exam and also their interest, they choose some study fields in a list and each volunteer waits to see the final results. Doubtlessly, perfect and correct information about universities will assist them in making a better decision (3, 4).

Several studies have been conducted in order to find the effective factors in selection of university fields all over the world. For instance, one survey conducted in Malaysian universities showed that previous knowledge about the course contents of the fields and information about future job opportunities had the most significant effect on selection of the study fields from the students' points of view (3, 4).

Another research performed in Canada in 2007 indicated the students' gender and parents’ level of education as the important factors (5). Moreover, numerous studies among the medical students have revealed some effective factors in the students’ intentions to select a university. These factors included gender, parents’ or spouse’s ideas, adequate future income, special field of study, quality of pedagogical program, the academic staffs adjacency of the university to their living place, and opportunity for research (5-9).

However, the meaning of selection will be more limited for the high school students in our country. They should pass the barrier of university entrance exam and then choose their study fields with regards to their ranks in the exam. Despite this restriction, if enough information about the universities over the country and the available study fields is given to a student, he/she will decide better and more confidently (10).

An investigation among the Iranian medical students in Jahrom (Fars province, south of Iran), for instance, showed that the students had a passive attitude toward their own fields of study. Overall, 49.7% of these selected university students had no tendency toward perusing their studies in medical sciences and about 54.1% stated that if they had another possibility to choose, they would select other fields, such as engineering, art, pharmacy, or dentistry (10). One other study which was done in Isfahan (center of Iran) showed the medical students’ negative attitude toward their future job opportunities. In fact, the students requested establishment of certain consultation and publicity centers about the rules, university fields' course contents, and future job opportunities all over the country in order to have a better insight for selection of their study fields (11).

In this regard, Shiraz University of Medical Sciences (SUMS), as one of the high rank universities in Iran, has been holding an exhibition called “University Open Day” in each summer since 2002 to introduce the university to high school students. Additionally, some university professors gave lectures in some selected Shiraz high schools in 2010-2011 and some student consultation centers were designed in 2012 to introduce the university to the students. Therefore, SUMS is one of the pioneers of publicity and spreading information among all the medical universities in Iran. In this way, not only it introduces its colleges, different fields, and equipment to high school student and helps them for better selection, but it also invites and accepts more talented students.

The designed informative methods of SUMS each have some advantages and disadvantages. Although exhibition and consulting, as the University Open Day, provide some information as well as more details on university facilities and fields, finding other notification methods seems necessary. At this point, the appropriate time for exhibition and efficient publicity is so important that it will need a perfect planning and management. 

A study conducted by Shayegh et al. in 2009 on the effectiveness of “University Open Day” program in the selection process of the study fields by the native students of SUMS showed the necessity for some revisions in this method (12).

The advantages of the university professors presenting lectures in high schools include being more economical and providing a suitable opportunity with no stress for the high school students to have face to face sessions with the professors before the entrance exam. However, due to the small number of interested volunteer professors, limited time, and large number of high schools, this method is done in some selected high schools in Shiraz. 

Finally, last summer (in 2012), university talented students office held a consultation exhibition for the students to choose their fields of study in the university. Some gifted students in each field introduced their study fields and assisted them to have a proper selection.

In the present research, we aimed to evaluate these programs among the undergraduate students who have entered our university in the recent 4 years (2009 to 2012) and asked about how they got familiar with the university and their consultation source for selection. The results of this research help improve the mentioned programs and design a new method for providing high-school students with enough and useful information about SUMS. In our research, we also evaluated the students’ level of satisfaction with their study fields as well as SUMS and asked them if they could suggest more suitable ways for introducing SUMS to other high school students. 

## Methods

The subjects of the present study included most university students in the field of medicine, dentistry, pharmacy and bachelor students in different fields in SUMS. The aim of this study was that census of most undergraduate and graduate students, professional students at the University to participate in a survey admitted at 2009-2012. The students were selected by stratified random sampling method (ten different colleges and entrance year from 1^st^ to 4^th^ are considered as the stratums).The number of participants is 2700 people and 2600 people have participated in the study. The fields of study include medicine, dentistry, pharmacy, nursing, midwifery, medical management, rehabilitation, paramedical sciences, and nutrition. The majority of the students were graduated in nursing and the minority were graduated in paramedical sciences and nutrition. Their demographic information, including gender, age, field of study, educational level, and entrance year, and the name of 6 cities with the most volunteers for SUMS have been recorded.

A verified questionnaire was designed according to the research objectives, distributed among the students, and then collected for analysis. The first question of the questionnaire was about the position of the present field of study (major) among the list of the fields in the entrance exam. The students’ answers were categorized into two parts including the 1^st^ choice to 10^th^ and the choices after 10th ones. This item was analyzed in different students according to their major and entrance year using Chi-square test.

The second question was about the students’ methods of familiarity with different fields of study in SUMS and the reasons for their selection. The following items were considered:  University open day exhibition, students' consultion center, university professors' lectures in high schools, visiting different colleges, adjacency to parents' living place, consultation with field selection institutes, consultation with teachers and/or family, SUMS website, newspapers, TV, radio, and the Internet, consultation with university professors and students, son or daughter of university scientific board.

For better evaluation of the students’ motivation and persistence for studying, they were asked to answer the question "*If you get an opportunity to select your field once more, would you select the same field and university*?" At the end, in order to assess the students’ motivation and insistence to follow their studies in SUMS, they were asked to assign a score of 0 to 10 to their study fields as well as their university (SUMS). This item was analyzed using ANOVA and t-test.

The validity of the questionnaire used in the text based on the objectives of the study has been funded by the research team. Reliability questions using Cronbach's alpha coefficient with a value of 0.75 has been approved. The SPSS for windows, version 14 (SPSS Inc, Chicago, IL, USA) was used for data analysis and 0.05 was considered as the significance level for statistical tests. 

## Results

The results are presented in tables and diagrams in different years. The present study was conducted on 2600 students comprising 85% of all the university students. Therefore, comparison of their answers with those of other students can be interesting. According to the results, the female to male ratio was 1.9 and all the university students were young (20.8±2.4 years old). The mean age of the female and male participants was 20.8±2.2 and 20.9±2.9 years, respectively and the difference was not statistically significant (p=0.23). Most of the participants were from the school of Nursing and Midwifery (23.4%) followed by the School of Medicine (17.5%). In this study, 25.9% of the students who had entered the university in 2012 were evaluated at the registration time. Among these students, 6.7% were living in Shiraz and other cities in Fars province, while 15.4% were living in the cities around Fars province. Therefore, totally, 76.1% of the university students were living with their parents in the cities around the university (Table 1). 

**Table 1:** Demographic information of the students participating in the study

**Gender**	Female	1690 ( 66 )
No. (%)	Male	910 (34)
**Age (Mean±SD)**	Female	20.8±2.4
	Male	20.9±2.9
**Field of study** **No. (%)**	Medicine	472 (17.5)
	Dentistry	193 (7.2)
	Pharmacy	192 (7.2)
	Nursing	629 (23.4)
	Midwifery	188 (7)
	Medical management	273 (10.1)
	Rehabilitation	248 (9.2)
	Paramedical health & nutrition	176 (6.5)
**Level of education** **No. (%)**	Bachelor of Science (BS)	1588 (61)
	Medical Doctor (MD)	1031 (39)
**Entrance year** **No. (%)**	2009	599 (39)
	2010	714 (27.5)
	2011	614 (23.6)
	2012	673 (25.9)
**The parents' living place** **No. (%) **	Fars province	753 (27.9)
	Yasuj	884 (32.8)
	Bushehr	112 (4.1)
	Kerman	66 (2.4)
	Tehran	63 (2.3)
	Ahvaz	52 (2)
	Isfahan	48 (1.8)
	Yazd	32 (1.2)

The first question of the questionnaire was about the position of the present field of study among the list of the fields in the entrance exam (Table 2). In this regards, no significant difference was found between the students who entered the university in 2012 and those entering the university in 2009-2011. Thus, the general acceptance rank of SUMS has been almost fixed during these years. Furthermore, the highest and lowest motivation for pursuing one's studies was observed in the school of medicine, paramedical sciences and management faculties, respectively. In the medical college, all the students had ranked their present field of study among the first 10 selected fields (Table 2). This shows the significant difference among the colleges (p<0.001).

**Table 2:** The position of the present accepted field (major) of study in the field selection list based on the year of entrance and field of study

	** The1st choice to 10^th^**	** The choices after 10^th^ ones **	p^*^
**Entrance year (%) **			0.998
2009	47.1	52.9	
2010	48.4	51.6	
2011	48.3	51.7	
2012	47.4	52.6	
**Field of study (%)**			<0.001
Medicine	100	0	
Dentistry	98.6	1.4	
Pharmacy	76.6	23.4	
Nursing midwifery	32.7	67.3	
Medical management	17.3	82.7	
Rehabilitation	22.1	77.9	
Paramedical	17.3	82.7	
Health and nutrition	24.4	75.6	

*Chi-square test

To detect the important factors affecting the students’ choice, the second question was about the students’ methods of familiarity with different fields of study in SUMS and the reasons for their choice.

According to the results in Table 3, family and teachers had a great impact on the students’ choice because more than half of the university students stated that their choice was the result of the recommendations given by their teachers and families. Adjacency to their parents' living place was also another important factor influencing their choice.

In Table 3, programs 1 to 4 have been planned by the educational experts of SUMS during the recent 10 years in order to introduce the university to high school students. As these plans and programs are mostly used by high school students living in Shiraz or Fars province, these items were investigated in local students. Low values obtained for these programs in Table 3 show their little effect on the local students’ choice. Therefore, it seems quite necessary to apply basic revisions to these plans.

**Table 3:** Students' familiarity method with SUMS and the reasons for their choices

**Options**	**Percent in Fars province native participants**	**Percent in total participant in evaluation**
University Open Day exhibition	12.1	0
Students' consultant center	6.4	0
University professors' lectures in high schools	5.5	0
Visiting different colleges	7.7	0
Adjacency to parents' living place	51.5	41.1
Consulting with field selection institutes	20.9	23.1
Consulting with teachers and/or family	61.5	52
SUMS website	15.4	12.1
Newspapers, TV, radio, and internet	13.2	20.4
Consulting with university professors and students	33	25.8
Son or daughter of university scientific board	4.4	6.5

Table 4 shows the students’ insistence on choosing of their major in SUMS. The highest score to SUMS was given by the first year students who had entered the university in 2012; this implies that there is a positive public view toward SUMS. This fact is so worthwhile. Nevertheless, the downward trend of the grade point average in the previous years shows the occasional decrease in the rate of satisfaction with the university. Therefore, further studies should be performed by the experts to find the reasons for this dissatisfaction.

Similar results were also obtained regarding grading the field of study. Very high mean scores among the newly entered students shows that most of the students were satisfied and liked their fields of study, especially at the beginning. This shows the students' high motivation to study in their majors. However, the downward trend of the grade point average is not so satisfactory.

**Table 4:** The score of students’ insistence on the choice of their university major in SUMS. Mean and standard deviation of the students’ scores to the university and their fields of study based on the entrance year. The score was classified between 0-10

**Grade**	**Entrance year**	**Mean±SD**	p*
**To Shiraz University of Medical Science (SUMS)**	2009	2.3±6.2	<0.001
	2010	2.0±6.7	
	2011	2.0±7.0	
	2012	1.4±8.9	
**To his/her own field of study**	2009	2.9±6.5	<0.001
	2010	2.6±7.0	
	2011	2.5±7.6	
	2012	0.6±9.5	

*ANOVA

The data of evaluation of the students’ insistence on selection of their major is expressed in Table 4. About 59.4% of the students gave positive answers to this question, which is highly valuable for the management of SUMS. In addition, 31.8% were satisfied with the university but not with their fields of study. Besides, 6.4% were dissatisfied with the university but not with their fields of study. Finally, 2% of the students were dissatisfied with both their fields of study and university. Dissatisfaction with SUMS and field of study increased little by little so that the results obtained among the students who had entered the university in 2009 (in the 4^th^ year of their study) showed nearly 16.3% dissatisfaction with both the university and the study fields (Table 5). Further studies are needed to be conducted in order to investigate the reasons for these results.

**Table 5:** The students' satisfaction with the university and their fields of study. The 1st to 4th year students were entered in 2012-2009

**Answers **(%)	**Year**			
	** 4^th^** **2009**	** 3^rd^** **2010**	** 2^nd^** **2011**	** 1^st^** **2012**
Same university and field	41.1	41.2	48.4	59.4
Same university but not same field	22	26.1	17.6	31.8
Same field buy not same university	18.8	19.2	21.2	6.7
Not this university and not this field	16.3	13.3	12.4	2

## Discussion

The present study aimed to find the most suitable methods to introduce SUMS to high school students. In this way, not only the university is introduced to high school students for an informed choice, but also the talented and motivated students are attracted to our university. As mentioned above, different methods have been designed by SUMS authorities during the recent years to reach this goal.

The aim of this study was to evaluate these methods and then improve and organize them if needed. Therefore, the role of other important factors, such as family, teachers, TV, radio, newspapers, field selection institutes, and university website was investigated, as well.

The results confirmed the necessity of some revisions and improvements on the designed programs to achieve the objectives. In addition, high school students were affected mostly by their families and teachers. Furthermore, adjacency of university to the parents' living place was an important factor in the students’ selection. These results indicate that family and teachers have great effects on the Iranian high school students’ important social decisions. Moreover, although Iranian university students are legally mature, they have strong emotional relationships with their families. Consequently, on designing the notification programs, the important role of family and high school teachers should be considered, as well. The more the students are informed, the more they will become familiar with the university and different fields of study and, consequently, will have a better choice.

According to the students’ suggestion, providing a complete pamphlet, including all the colleges and study fields, future vocational prospect in each field, and the rank to be accepted in a certain field is necessary for decision making. Mentioning all the capabilities, grants, and the present rank of the university among the universities all over the country and even the world can also be helpful. This report can be printed or prepared on CDs and distributed among the senior high school students in Shiraz as well as Fars province. In addition, improvement of SUMS website helps to introduce the university to all national and international students.

At the end, to evaluate the level of educational motivation in SUMS, the students were asked to rank SUMS and their fields of study. The high grade among the newly entered university students shows the good view of the university in the society and also the students' high motivation for studying in this university. However, the downward trend of these grades among the senior university students should be investigated in future studies in order to find the reasons. 

Most of the participants in the present study were female. There are reports which explain that women are more concerned with uncertainty, doubts, and the dynamism that influence decision making. These researchers put more emphasis on time and money. They were also more concerned about the outcomes of the decision, no matter whether these affected them or other people. Conversely, men assigned more importance to determination of the goals of the decision. Generally, men are more motivated during the decision processing and also feel more intensely the pressure from all the work-related aspects (13-15).

Considering the participants’ age, it should be noted that medical students are newly graduated from high school and have no premedical major as biological sciences. This can affect the university decision. With regard to age, Sanz de Acedo et al. (2007) indicated that the youth felt significant pressure from emotional and social aspects of their decisions, while the adults felt this pressure to a lesser extent. One explanation for these findings could be that the majority of the youth lack sufficient knowledge and experience in certain decision areas (13).

Overall, providing the high school students all over the country with adequate information about the nature of the study fields and universities’ facilities may bring about deep insight and knowledge for them. This, in turn, leads to an informed field choice and increased motivation for studying in universities. Hence, more detailed studies are recommended to be performed on the issue in future (14, 15).

## Conclusion

In the current study, the most significant references for the students' academic field selection were high school teachers and the students' parents. Moreover, the adjacency of university to one's living place was reported as the most significant factor in this regard. Accordingly, the new informative method should consider the important role of high school teachers and families in the students’ decision making. Furthermore, preparing an illustrative report from the important features of the university, such as the colleges, study fields, and future vocational prospects in each field, should also be taken into account. 

## References

[1] Chuang NK, Walker K, Natalie CB (2009). Student perceptions of career choices: the impact of academic major. Journal of Family & Consumer Sciences Education.

[2] Aycan Z, Fikret-Pasa S (2003). Career choices, job selection criteria, and leadership preferences in a transitional nation: The case of Turkey. Journal of Career Development.

[3] Misran N, Nurhanum Syed Sahuri S, Arsad N, Hussain H, Diyana Wan Zaki WM, Abd Aziz N (2012). Malaysian matriculation student’s factors in choosing university and undergraduate program. Asian Social Science.

[4] Ming JSK (2010). Institutional factors influencing college students choice decision in Malaysia: A conceptual framework. International Journal of Business and Social Science.

[5] Boudarbat B, Montmarquette C (2009). Choice of fields of study of Canadian University graduates: The role of gender and their parents’ education. Education Economics.

[6] Rehman A, Rehman T, Shaikh MA, Yasmin H, Asif A, Kafil H (2011). Pakistani medical students’ specialty preference and the influencing factors. J Pak Med Assoc.

[7] Saeed S, Jimenez M, Howell H, Karimbux N, Sukotjo C (2008). Which factors influence students’ selection of advanced graduate programs? One institution’s experience. J Dent Educ.

[8] Wang T, Wong B, Huang A, Khatri P, Ng C, Forgie M (2011). Factors affecting residency rank-listing: a Maxdiff survey of graduating Canadian medical students. BMC Med Educ.

[9] Aagaard EM, Julian K, Dedier J, Soloman I, Tillisch J, Pérez-Stable EJ (2005). Factors affecting medical students' selection of an internal medicine residency program. J Natl Med Assoc.

[10] Iravani K, Amini M, Doostkam A (2002). A survey of medical students' attitude toward medicine and its future in Jahrom medical university (basic and clinical stage). IJME.

[11] Sadr Arhami N, Kalantari S, Atarod S (2004). Medical students' attitude towards their field of study and future career. IJME.

[12] Shayegh S, Mirkhani H, Bazrafkan L (2009). Investigation of Shiraz University of Medical Sciences students view about the effectiveness of University Open Day. Strides in Development of Medical Education.

[13] Sanz de Acedo RML, Sanz de Acedo BMT, Cardelle-Elawar M (2007). Factors that affect decision making: gender and age difference. International Journal of Psychology and Psychological Therapy.

[14] Aloka PJO, Bojuwoye O (2013). Gender, Age and Teaching Experiences Differences in decision-making behaviors of members of selected Kenyan secondary school disciplinary panels. Asian Social Science.

[15] Haris I (2012). Determinant factors of decision making process in higher education institution (A case of state University of Gorontalo, Indonesia). Global Journal of Management and Business Research.

